# RPL28 mediates sorafenib resistance in hepatocellular carcinoma by downregulating CDC6 expression

**DOI:** 10.3389/fonc.2026.1741406

**Published:** 2026-02-16

**Authors:** Yi Shi, Fangfang Chen, Yuanyuan Weng, Hang Zeng, Gang Chen

**Affiliations:** 1 Department of Molecular Pathology, Clinical Oncology School of Fujian Medical University, Fujian Cancer Hospital, Fuzhou, Fujian, China; 2 Department of Pathology, School of Basic Medical Sciences, Fujian Medical University, Fuzhou, Fujian, China

**Keywords:** ribosomal proteins L28 (RPL28), sorafenib resistance, hepatocellular carcinoma, CDC6, transcriptome, proteome

## Abstract

**Aim:**

Sorafenib is a milestone targeted therapy for advanced hepatocellular carcinoma (HCC), yet resistance to this agent severely limits its clinical efficacy. The molecular mechanisms underlying sorafenib resistance are incompletely understood. Ribosomal proteins (RPs) have been increasingly implicated in cancer progression and drug resistance, but the role and mechanism of ribosomal protein L28 (RPL28) in sorafenib resistance in HCC remains unexplored.

**Methods:**

We investigated the functional role of RPL28 in sorafenib-resistant HCC using HepG2 and HCCLM3 cell models. RPL28 was silenced by siRNA, and effects on cell proliferation, and migration were assessed by CCK-8 and migration assays. Integrated transcriptomic and proteomic analyses were performed to delineate downstream pathways. The expression of immune-related proteins and key targets was validated by Western blotting.

**Results:**

RPL28 expression was significantly reduced at both mRNA and protein levels in knockdown cells of sorafenib-resistant HepG2 and HCCLM3. RPL28 knockdown inhibited proliferation and migration in resistant HCC cells. Transcriptomic and proteomic analyses identified CDC6 as a key downstream target of RPL28. CDC6 expression was consistently decreased in RPL28 KD cells, while EGFR and TRAF6 remained unchanged. GO and KEGG pathway enrichment revealed that RPL28 modulates pathways involved in DNA replication, immune regulation, and metabolic adaptation. Notably, no significant changes were observed in MHC-I and PD-L1 expression following RPL28 knockdown.

**Conclusions:**

Our findings demonstrate that RPL28 contributes to sorafenib resistance in HCC by upregulating CDC6, contributing to tumor proliferation and drug resistance. The newly identified RPL28-CDC6 axis represents a novel mechanism of resistance and a potential therapeutic target to overcome treatment limitations in HCC.

## 1 Introduction

Hepatocellular carcinoma (HCC) is one of the most common and deadly types of liver cancer worldwide (1). Sorafenib was the first molecularly targeted drug proven to have significant efficacy against HCC, marking a milestone in its treatment (2, 3). Although new drugs of the same class have been introduced in recent years, narrowing the clinical application of sorafenib, it remains an important targeted agent for advanced HCC, particularly in resource-limited settings or in patients who are intolerant to other therapies. This includes patients with a history of severe hypertension, risk of gastrointestinal bleeding, or those who are elderly, underweight, or have poor baseline conditions (4, 5). However, both primary and acquired resistance to sorafenib significantly limit its clinical efficacy and contribute to poor patient prognosis (6). In recent years, genomic and proteomic studies have begun to reveal potential molecular mechanisms underlying resistance, but the specific factors responsible for sorafenib resistance have not yet been fully elucidated. This remains one of the major obstacles to improving treatment outcomes and patient prognosis (7).

Ribosomal protein L28 (RPL28), a component of the 60S ribosomal subunit, has recently been implicated in tumor development and chemo-resistance beyond its traditional role in translation (8). Several ribosomal proteins have been shown to regulate oncogenic pathways and influence drug sensitivity (9). Our preliminary studies suggest that elevated expression of RPL28 is closely associated with sorafenib resistance in HCC, although the underlying mechanisms remain unclear (10). Meanwhile, cell division cycle 6 protein (CDC6), a key factor in DNA replication licensing, is believed to play a significant role in tumor cell proliferation and drug resistance (11, 12). However, whether RPL28 exerts its function in HCC by regulating CDC6, and how this interaction is related to sorafenib treatment, has not yet been investigated or reported.

Given these considerations, we hypothesized that RPL28 may be a critical mediator of sorafenib resistance in HCC through modulation of CDC6 expression. This study was designed to investigate the expression and functional consequences of RPL28 knockdown in HCC cell lines. Through transcriptomic and proteomic analyses, we aimed to delineate the downstream pathways affected by RPL28 and identify its role in cellular proliferation, migration, and immune modulation. By focusing on the RPL28-CDC6 axis, we aimed to identify a novel regulatory mechanism of sorafenib resistance, providing a potential biomarker or therapeutic target for HCC treatment.

In this study, we used siRNA-mediated knockdown to suppress RPL28 expression in HepG2 and HCCLM3 cells. Functional assays including cell proliferation, and migration were performed. Furthermore, transcriptomic and proteomic profiling were used to assess global changes in gene and protein expression. Special attention was given to changes in CDC6 and immune-related pathways. Our results reveal that RPL28 silencing downregulates CDC6, suggesting that the RPL28-CDC6 regulatory axis is a key driver of therapeutic resistance in HCC.

## 2 Materials and methods

### 2.1 Cell lines and culture, RPL28 knockdown, RNA extraction and qRT-PCR and western blotting

The human sorafenib-resistant HCC cell lines HepG2 and HCCLM3 were obtained from [CTCC-0105-NY and CTCC-0541-NY] and cultured in Dulbecco’s Modified Eagle Medium (DMEM) supplemented with 10% fetal bovine serum (FBS), 100 U/mL penicillin, and 100 μg/mL streptomycin. The maintenance concentration of sorafenib (MCE, BAY 43-9006) for drug resistance is 8 μm for HepG2, and 20μm for HCCLM3. Cells were maintained at 37°C in a humidified atmosphere containing 5% CO_2_. All cell lines were authenticated by short tandem repeat (STR) profiling and confirmed to be free of mycoplasma contamination.

Small interfering RNA (siRNA) targeting RPL28 and a negative control siRNA (NC) were purchased from GeneChem Co., Ltd. Transfections were performed using E-trans (GeneChem) according to the manufacturer’s protocol. Knockdown efficiency was verified by quantitative real-time PCR (qRT-PCR) and Western blotting 96 hours post-transfection. RPL28 antibody was purchased from Abcom and β-actin was loading control (13, 14).

### 2.2 Cell proliferation and viability assays, cell migration assay, and immune-related proteins detection

Cell proliferation was assessed using the CCK-8 assay (Dojindo). Transfected cells were seeded into 96-well plates and absorbance at 450 nm was measured daily for 5 days. Cell migration was measured using the Celigo imaging cytometer system. Transfected cells were seeded in 96-well plates and imaged for 0hour, 24hours, 48hours. The migration rate was calculated based on confluency changes over time using Celigo software. Immune-related protein, PD-L1 (CST) and MHC Class I(CST) detection was conducted by western blotting (15–17).

### 2.3 Transcriptome sequencing and proteomic analysis

Total RNA was extracted from NC and RPL28 KD cells and subjected to library preparation using NEBNext^®^ UltraTM RNA Library Prep Kit and sequenced on an Illumina platform. Differential expression analysis was performed using DESeq2. Genes with adjusted *P* < 0.05 and |log_2_ fold change| > 1 were considered significantly differentially expressed. GO and KEGG enrichment analyses were conducted using the clusterProfiler package in R.

Protein samples were digested and analyzed on a nanoElute (Bruker, Bremen, Germany) coupled to a timsTOF Pro (Bruker, Bremen, Germany) equipped with a CaptiveSpray source. Differential protein expression was determined using PaSER2023. Analyses were performed using uniprot_homo_20231008_20427_9606_swiss_prot databases. (http://www.uniprot.org).

All experiments were performed in triplicate. Data are presented as mean ± standard deviation (SD). Comparisons between two groups were performed using unpaired two-tailed Student’s *t*-test. *P* < 0.05 was considered statistically significant.

### 2.4 Integrated transcriptomic and proteomic analysis

Transcriptome sequencing provides information on gene expression, while proteome sequencing reveals data on protein expression and post-translational modifications. The levels of gene transcription and protein expression are not always fully consistent, as post-transcriptional RNA regulation and factors such as translation, modification, and degradation can influence protein abundance. Integrated transcriptomic and proteomic analysis is based on the standard differential analysis results of each omics layer, combining differentially expressed genes and proteins. By correlating the two datasets through GO functional annotation, KEGG pathway enrichment, and expression correlation analyses, key genes, key proteins, and critical signaling pathways can be identified.

### 2.5 Western blot analysis of RPL28 downstream targets

According to integrated analysis, Western blot validated of downstream functional targets of RPL28. Cells were lysed with RIPA buffer supplemented with protease inhibitors. Equal amounts of protein were separated via SDS-PAGE and transferred to PVDF membranes. Membranes were blocked with 5% non-fat milk and incubated overnight at 4°C with primary antibodies against CDC6 (CST), EGFR (Maxim), TRAF6 (CST) and β-actin (loading control). After incubation with HRP-conjugated secondary antibodies, bands were visualized using ECL reagents (Cell signaling technology) and quantified using Image software.

## 3 Results

### 3.1 RPL28 knockdown reduces its expression in sorafenib-resistant HCC Cells

**Figure 1:**
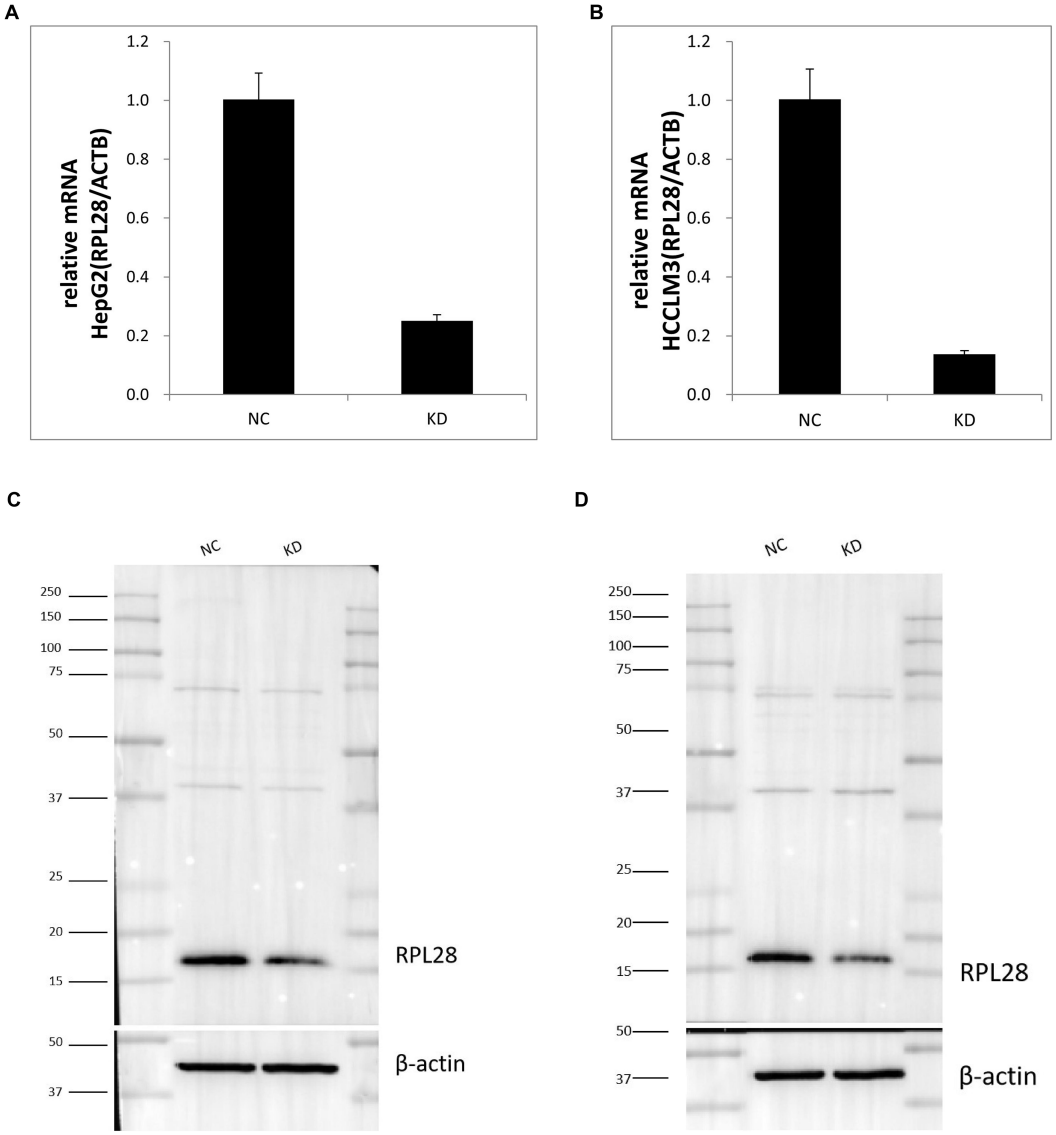
RT-PCR and Western blotting analysis. Compared to negative control (NC) cells. **(A, B)** RPL28 mRNA levels in both HepG2 and HCCLM3 KD cells; **(C, D)** RPL28 protein in KD groups for HepG2 and HCCLM3 cell lines.

To investigate the role of RPL28 in sorafenib-resistant HCC, we first established RPL28 knockdown (KD) models in sorafenib-resistance HepG2 and HCCLM3 cells. RT-PCR analysis revealed a significant reduction in RPL28 mRNA levels in both HepG2 and HCCLM3 KD cells compared to negative control (NC) cells (**Figures 1A, B**). Consistent with mRNA data, Western blotting confirmed the marked decrease of RPL28 protein in KD groups for both cell lines (**Figures 1C, D**), with quantification shown in (**Supplementary Figures 1A, B**). These results confirm efficient knockdown of RPL28 at both transcript and protein levels.

### 3.2 RPL28 knockdown inhibits cell proliferation and migration, no regulates immune-related protein expression

**Figure 2:**
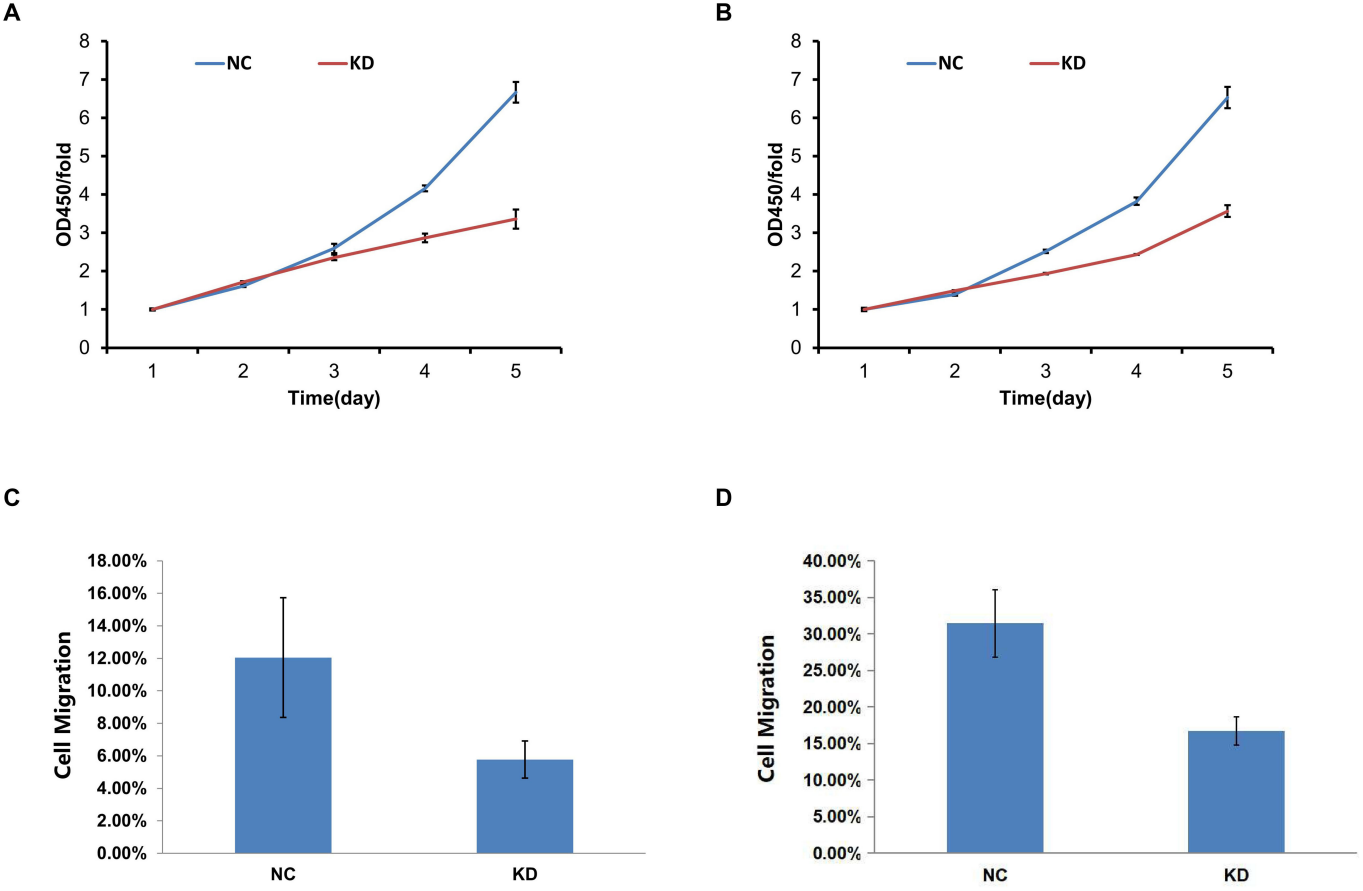
**(A, B)** CCK-8 assays analyzed the proliferation rate of sorafenib-resistant HepG2 and HCCLM3 cells over a 5-day period upon KD of RPL28; **(C, D)** Celigo migration assays demonstrated cell migration in KD cells compared to NC.

To evaluate the functional consequence of RPL28 silencing, CCK-8 assays were conducted. As shown in (**Figures 2A, B**), KD of RPL28 significantly reduced the proliferation rate of sorafenib-resistant HepG2 and HCCLM3 cells over a 5-day period. Additionally, Celigo migration assays demonstrated decreased cell migration in KD cells compared to NC (**Figures 2C, D**), indicating that RPL28 contributes to sorafenib-resistance HCC cell growth and motility.

Given the relevance of immune modulation in drug resistance, we analyzed the expression of MHC Class I and PD-L1 in RPL28 KD sorafenib-resistant cells. Western blot results showed that there was no change in the expression of MHC-I and PD-L1 molecules in both HepG2 and HCCLM3 KD sorafenib-resistant cells compared to controls (**Supplementary Figures 2A, B**).

### 3.3 Transcriptomic and proteomic profiling reveals downregulation of CDC6 upon RPL28 knockdown

**Figure 3:**
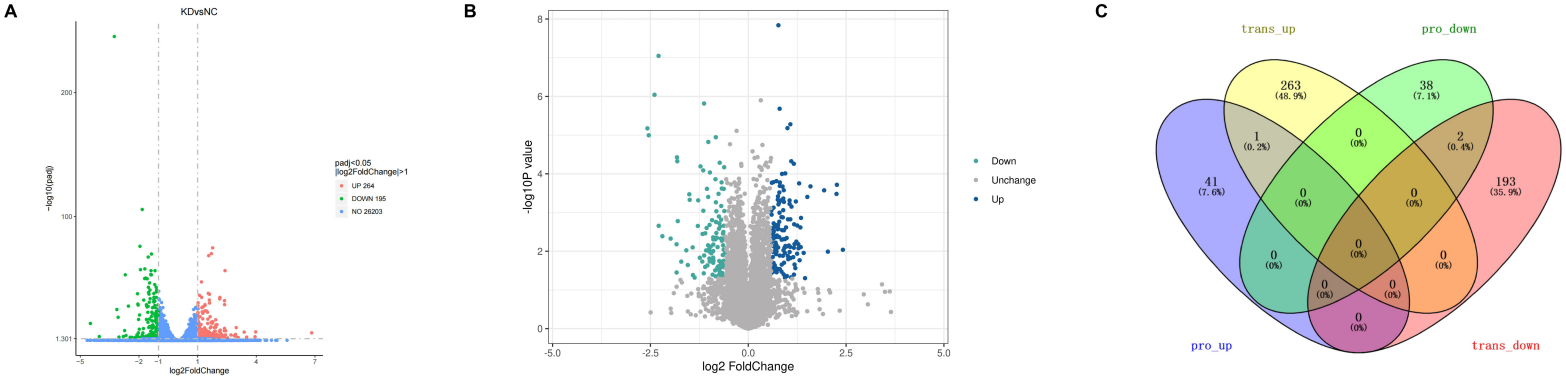
**(A)** The volcano plot of RNA sequencing on RPL28 knockdown (KD) and control (NC) sorafenib-resistant hepatocellular carcinoma (HCC) cells; **(B)** The volcano plot of comparative proteomic analysis on both cells; **(C)** The Venn diagram of integrative analysis between the transcriptome and proteome.

To determine the transcriptional alterations associated with sorafenib resistance, RNA sequencing was performed on RPL28 knockdown (KD) and control (NC) sorafenib-resistant HCC cells. As shown in the volcano plot (**Figure 3A**), a total of 459 genes were significantly differentially expressed, including 264 upregulated and 195 downregulated genes (adjusted *P* < 0.05, |log_2_FoldChange| > 1). The vast majority of transcripts remained unchanged (*n* = 26,414), indicating specific transcriptional modulation rather than global shifts in gene expression. And to explore the proteomic alterations underlying sorafenib resistance, we performed comparative proteomic analysis between sorafenib-resistant HCC cells. As shown in the volcano plot (**Figure 3B**), 430 proteins were significantly upregulated (blue dots) and 359 proteins downregulated (green dots) in KD cells, while the majority remained unchanged (gray dots). These results indicate that sorafenib resistance in HCC is associated with specific proteomic alterations.

**Figure 4:**
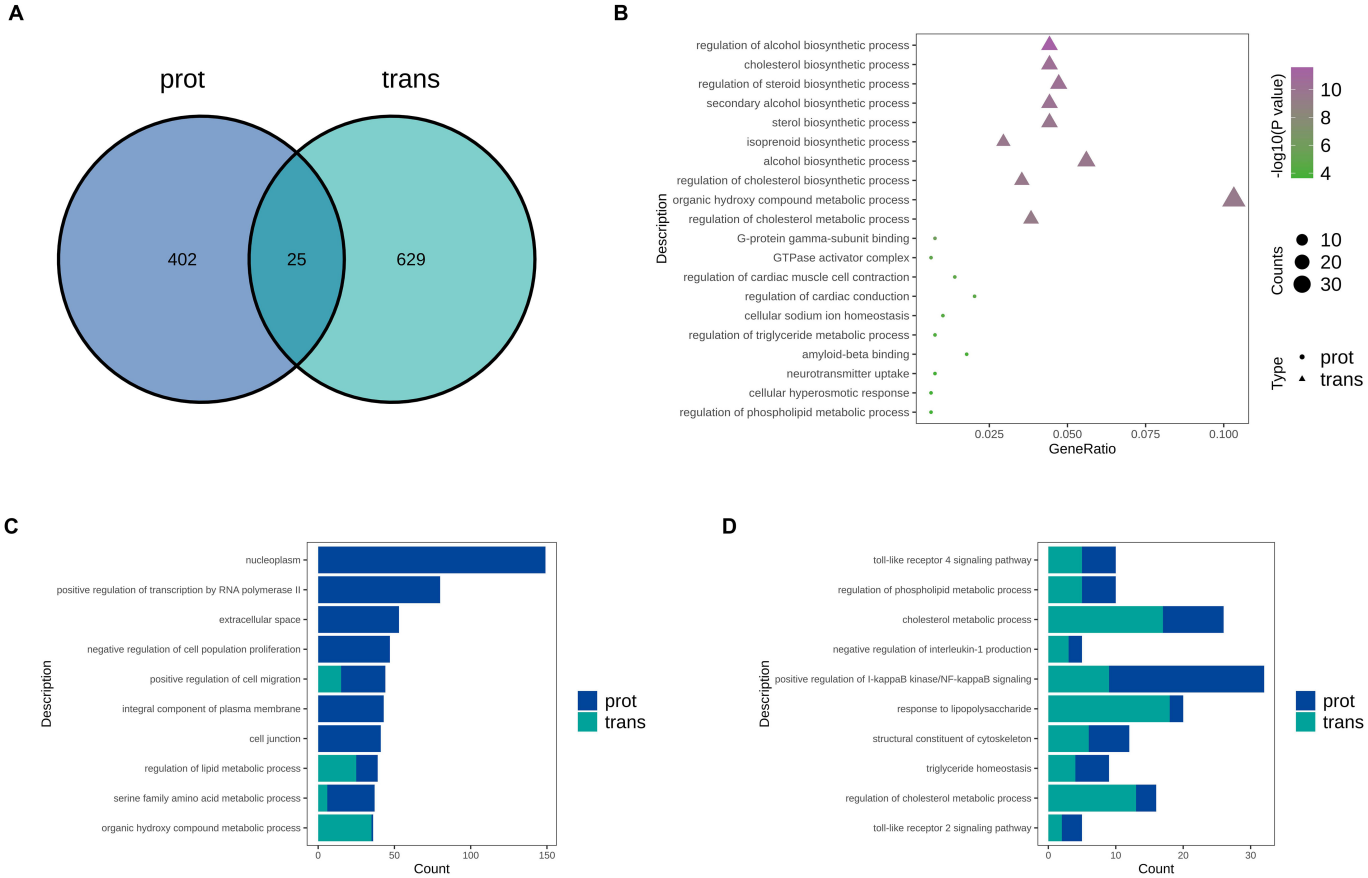
**(A)** GO Term Venn Diagram: the number in the overlapping region of the Venn diagram represents the number of GO terms commonly enriched in both omics datasets; the numbers in the non-overlapping regions represent GO terms uniquely enriched in the proteomic and transcriptomic datasets, respectively. **(B)** GO Term Bubble Plot: differentially expressed molecules from the proteomic and transcriptomic datasets were subjected to GO enrichment analysis. GO terms were ranked by ascending p-value, and the top 10 most significant terms from each dataset were selected, yielding up to 20 GO terms in total. **(C)** Top 10 GO Term Bar Chart: the numbers of differentially expressed mRNAs and proteins enriched in each GO term were combined. The top 10 GO terms with the largest total number of differentially expressed molecules were selected for display. **(D)** Common GO Term Bar Chart: among the GO terms enriched in both datasets, the top 10 most significant common GO terms were selected for display.

To identify the biological processes commonly involved at both the transcriptomic and proteomic levels, we performed an integrative analysis. As shown in the Venn diagram (**Figure 3C**), there were relatively few overlapping differentially expressed genes between the transcriptome and proteome, suggesting significant post-transcriptional regulation. Notably, RPL28 and GPAM were both downregulated at both the transcript and protein levels. Among them, RPL28 was the most significantly downregulated molecule at both levels, which is consistent with our experimental design and validates the accuracy of our model. GO functional Venn analysis revealed 25 shared GO terms between the two datasets (**Figure 4A**), indicating some consistency in functional impacts. However, this limited overlap suggests that while there are commonalities in biological responses at the transcriptional and translational levels, there are also distinct differences, highlighting the importance of multi-omics integrative analysis. The GO bubble plot (**Figure 4B**) illustrates significantly enriched biological processes in each omics layer. In the transcriptome, genes were mainly enriched in metabolism-related pathways such as cholesterol and steroid biosynthesis, while the proteome showed enrichment in GTPase activity, neurotransmitter regulation, and cellular sodium ion homeostasis. However, the TOP10 GO bar chart (**Figure 4C**) revealed significant enrichment in omics for processes including nucleoplasm, transcriptional regulation by RNA polymerase II, cell migration, and lipid metabolic processes. These findings suggest that RPL28 may play a crucial role in regulating gene expression and remodeling the tumor microenvironment. Among the commonly enriched GO terms (**Figure 4D**), inflammation- and immune-related pathways were particularly prominent. Additionally, metabolic pathways such as cholesterol and phospholipid metabolism were also jointly enriched, suggesting that RPL28 may mediate drug resistance through mechanisms involving metabolic reprogramming and immune evasion.

**Figure 5:**
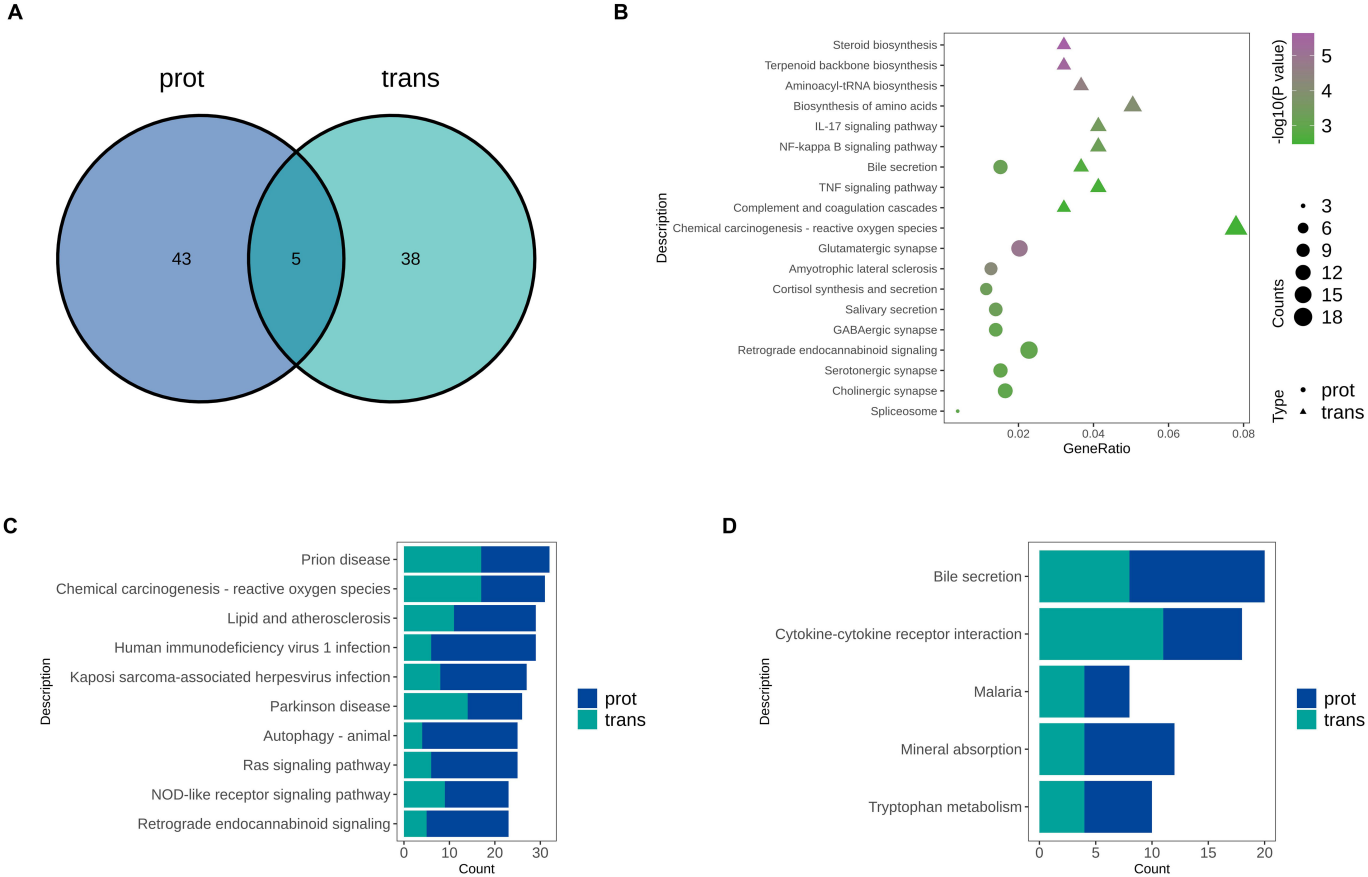
**(A)** KEGG Pathway Venn Diagram: the number in the overlapping region of the Venn diagram represents the number of KEGG pathways commonly enriched in both omics datasets; the numbers in the non-overlapping regions represent KEGG pathways uniquely enriched in the proteomic and transcriptomic datasets, respectively. **(B)** KEGG Pathway Bubble Plot: differentially expressed molecules from the proteomic and transcriptomic datasets were subjected to KEGG pathway enrichment analysis. Pathways were ranked by ascending p-value, and the top 10 most significant pathways from each dataset were selected, yielding up to 20 pathways in total. **(C)** Top 10 KEGG Pathway Bar Chart: the numbers of differentially expressed mRNAs and proteins enriched in each KEGG pathway were combined. The top 10 KEGG pathways with the largest total number of differentially expressed molecules were selected for display. **(D)** Common KEGG Pathway Bar Chart: common KEGG pathways are visually presented in a bar chart.

To further investigate the potential mechanisms by which RPL28 regulates sorafenib resistance in HCC, we performed KEGG pathway enrichment analysis on both transcriptomic and proteomic datasets. As shown in (**Figure 5A**), only five KEGG pathways were commonly enriched between the transcriptome (trans) and proteome (prot) data. This limited overlap suggests significant post-transcriptional regulation and divergence between transcriptional and translational pathway involvement during the acquisition of resistance. The KEGG bubble plot (**Figure 5B**) reveals distinct enrichment patterns between the two datasets: transcriptomic data were primarily enriched in metabolism- and inflammation-related pathways, whereas proteomic data were predominantly enriched in neurotransmitter signaling and immune response pathways. These results indicate that RPL28 knockdown induces complex reprogramming of various biological processes. The TOP10 pathway bar chart (**Figure 5C**) further demonstrates that both omics levels show significant enrichment in immunoregulation, viral infection, and lipid metabolism pathways. (**Figure 5D**) highlights five KEGG pathways that are commonly enriched in both datasets, including bile secretion, cytokine–cytokine receptor interaction and tryptophan metabolism. The co-enrichment of these pathways suggests that immune regulation and metabolic adaptation may be key components of the resistance mechanism. In particular, the “cytokine–cytokine receptor interaction” pathway implies the existence of immune evasion mechanisms, while “bile secretion” and “tryptophan metabolism” further support the presence of metabolic reprogramming—hallmark features of drug-resistant cancer cells.

### 3.4 CDC6 is a downstream target of RPL28CDC

**Figure 6:**
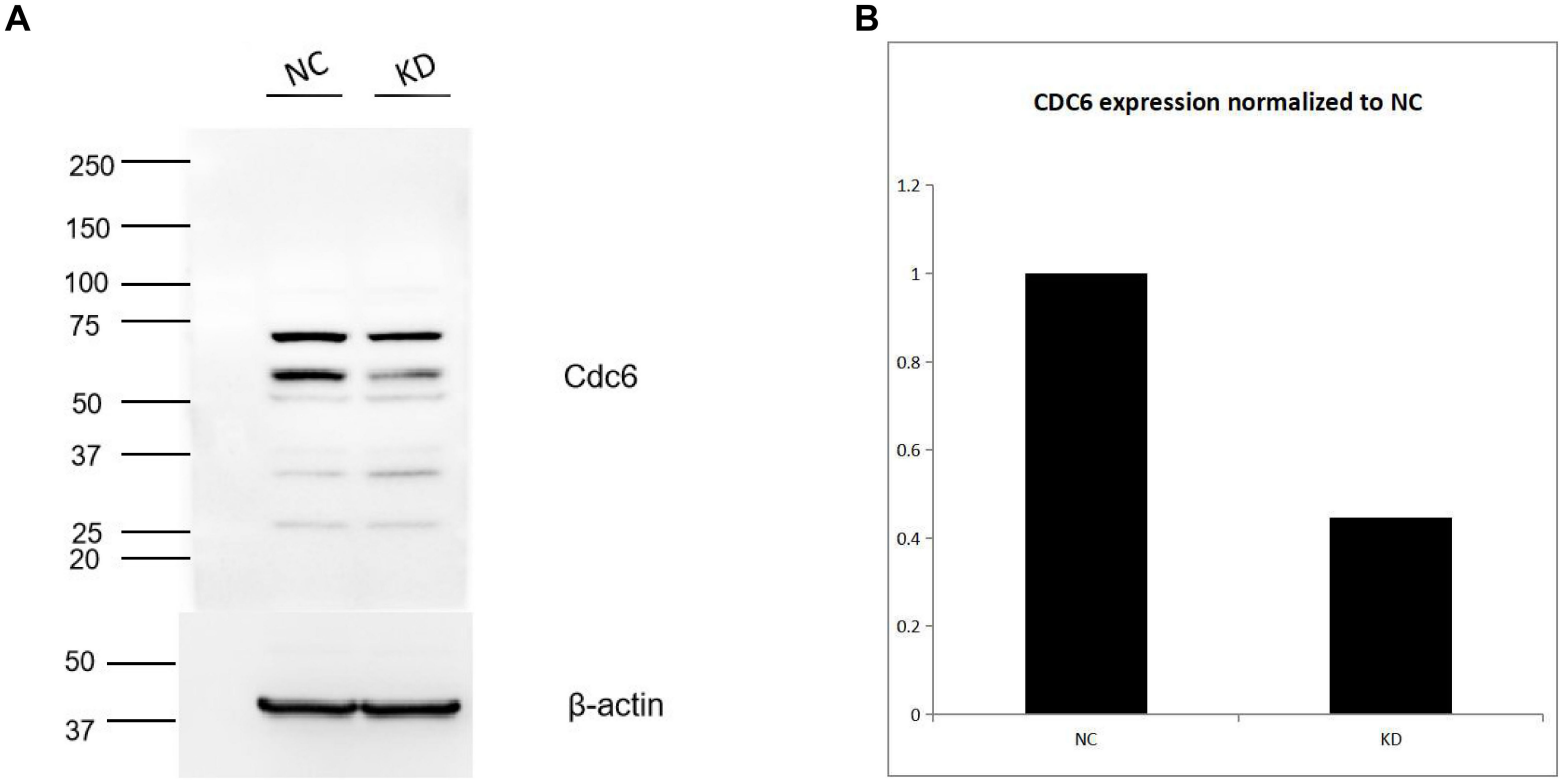
**(A)** Western blotting analysis CDC protein in RPL28 KD sorafenib-resistant cells. **(B)** densitometric quantification of CDC protein.

Among the downregulated targets, CDC6 was consistently decreased in RPL28 KD sorafenib-resistant cells at both mRNA and protein levels. Western blot analysis confirmed reduced CDC6 protein expression (**Figure 6A**), with densitometric quantification demonstrating a significant reduction in the KD group (**Figure 6B**). In contrast, expression of EGFR and TRAF6 was not significantly altered (**Supplementary Figures 2C, D**), indicating specificity of CDC6 regulation. These results suggest that RPL28 promotes HCC growth and sorafenib resistance, at least in part, through upregulation of CDC6.

## 4 Discussion

### 4.1 Summary of key results

In this study, we identified RPL28 as a mediator of sorafenib resistance in HCC through the regulation of CDC6. Our experiments demonstrated that RPL28 is highly expressed in HCC cells, and its knockdown significantly inhibits cell proliferation and migration. CDC6, a crucial DNA replication regulator, was downregulated at both mRNA and protein levels after RPL28 knockdown. These findings suggest a mechanistic link between RPL28 and CDC6 expression, potentially underlying the observed resistance to sorafenib in HCC cells.

### 4.2 Interpretation and literature comparison

Our findings align with growing evidence of RPs extra-ribosomal functions in cancer.

For instance, RPL11 and RPL5 have been shown to regulate p53 activity (18), while RPL22L1 has been implicated in epithelial-mesenchymal transition (EMT) in ovarian cancer (19). Our findings extend this paradigm to RPL28, which had previously been underexplored in the context of HCC. By biological and omics approaches, our results suggest that RPL28 mediates sorafenib resistance in HCC by regulating the CDC6 molecule. Prior studies have implicated factors such as AKT/mTOR signaling, BCL2-family proteins, and hypoxia-inducible factors in resistance mechanisms (20, 21). Unlike these mechanisms, RPL28 appears to regulate sorafenib response via CDC6 - a DNA replication licensing factor essential for S-phase entry. Our data showed a consistent reduction in CDC6 expression following RPL28 silencing, suggesting that the suppression of replication licensing may limit cell cycle progression and enhance sorafenib efficacy.

CDC6 overexpression has been associated with various malignancies and is known to promote chemoresistance through cell cycle dysregulation and apoptosis inhibition (22). Our study suggests that RPL28 may act upstream to support CDC6 stability or transcription. This connection underscores a non-canonical role of ribosomal proteins in gene regulation. Notably, we did not observe significant changes in other candidate proteins like EGFR or TRAF6, highlighting a degree of specificity in the RPL28-CDC6 axis. Therefore, we propose two hypotheses: A.RPL28 induces a drug-resistant quiescent state or activates alternative survival pathways by suppressing CDC6 and causing cell cycle stress. GO enrichment terms such as “negative regulation of cell population proliferation” suggest perturbation in cell cycle regulation. Thus, RPL28 knockdown leads to decreased CDC6 expression, resulting in G1/S arrest or replication stress, which may trigger adaptive responses in tumor cells, including activation of metabolic reprogramming or drug resistance pathways. B.RPL28 suppresses CDC6 and subsequently activates the NF-κB pathway, inducing inflammatory factors that enhance immune suppression in the tumor microenvironment and promote drug resistance. GO analyses showed enrichment in immune-inflammatory pathways such as “positive regulation of I-kappaB kinase/NF-kappaB signaling” and “interleukin-1 production.” the RPL28-CDC6 axis may regulate NF-κB activity, promoting tumor cell survival through inflammation-induced resistance mechanisms and upregulation of pro-inflammatory mediators. While the exact regulatory mechanism remains to be elucidated, our proteomic and WB results support a strong and specific association between RPL28 activity and CDC6 expression.

Collectively, our results present a cohesive narrative in which RPL28 upregulation supports HCC cell proliferation and promotes sorafenib resistance via CDC6. This functional axis has not been previously described in the context of liver cancer or sorafenib treatment. Although prior literature has established RPL28 as a ribosomal component with limited regulatory roles, our study presents evidence for its drug-resistance-promoting effects, thus challenging the traditional view of ribosomal proteins as passive players in cancer biology. Importantly, this work fills a significant gap in our understanding of sorafenib resistance and identifies RPL28-CDC6 as a potential therapeutic target.

### 4.3 Academic contributions and significance

The primary contribution of this study is the identification of the RPL28-CDC6 axis as a novel mechanism mediating sorafenib resistance in HCC. By integrating functional assays with transcriptomic and proteomic analyses, we demonstrated that RPL28 not only supports tumor cell proliferation and migration but also modulates key pathways involved in DNA replication and immune evasion. This study is among the first to propose a mechanistic link between a ribosomal protein and CDC6 expression in the context of drug resistance. These insights not only deepen our understanding of HCC pathophysiology but also propose RPL28 as a potential biomarker for therapy selection and a candidate for combination treatments with immune checkpoint inhibitors or targeted therapies.

### 4.4 Limitations and future directions

While our study provides compelling evidence linking RPL28 to sorafenib resistance, several limitations must be acknowledged. First, our experiments were conducted *in vitro* using two HCC cell lines. Although these models are widely used and representative, they may not fully recapitulate the heterogeneity and complexity of patient tumors. Future work should include validation of RPL28 and CDC6 expression patterns in primary HCC tissues and patient-derived xenograft (PDX) models. Second, the precise regulatory mechanism remains unclear. Further studies employing chromatin immunoprecipitation, promoter assays, or proteasome inhibition are needed to determine whether RPL28 affects CDC6 at the transcriptional or post-translational level. Another limitation lies in the exclusive focus on sorafenib. Given that lenvatinib and other tyrosine kinase inhibitors (TKIs) are now approved for HCC, future work should test whether RPL28 modulates resistance to these agents as well. Considering the shared downstream signaling pathways among TKIs used in HCC treatment, it will be of particular interest to investigate whether the RPL28–CDC6 axis represents a common determinant of TKI resistance beyond sorafenib.

Although transcriptomic and proteomic analyses revealed alterations in immune-related pathways following RPL28 knockdown, we did not observe significant changes in the expression of key immune checkpoint molecules such as MHC-I and PD-L1 in sorafenib-resistant HCC cells. This suggests that while RPL28 may influence the tumor immune microenvironment indirectly through inflammatory or signaling pathways, it may not directly modulate classical antigen presentation or immune evasion markers. As such, further investigation using *in vivo* models or co-culture systems with immune cells is required to clarify the immunological consequences of RPL28 regulation and to determine whether RPL28-mediated resistance involves alternative immune evasion strategies beyond MHC-I or PD-L1 modulation. Despite ongoing challenges, our study provides a solid foundation for understanding the role of ribosomal proteins in tumor drug resistance and opens new avenues for therapeutic strategies in HCC. Given the absence of immune cells in the current experimental system, the observed immune-related pathway changes should be interpreted with caution and primarily regarded as hypothesis-generating.

## 5 Conclusion

This study identifies RPL28 as a key contributor to sorafenib resistance in HCC by upregulating CDC6 and promoting tumor growth. RPL28 knockdown sensitized cells to sorafenib and suppressed CDC6 expression. These findings reveal a novel RPL28-CDC6 axis and highlight RPL28 as a potential target to improve HCC treatment outcomes.

During the preparation of this work, the author(s) used Chatgpt4.o in order to polish language. After using this tool, the author(s) reviewed and edited the content as needed and take(s) full responsibility for the content of the publication.

## Data Availability

Due to compliant restrictions, the raw data cannot be made publicly available. However, de-identified data may be obtained from the corresponding author upon reasonable request.

## Supplementary material

Supplementary Figure 1 The quantification of RPL28 protein. **(A)** HepG2 KD sorafenib-resistant cells; **(B)** HCCLM3 KD sorafenib-resistant cells.

Supplementary Figure 2 Western blot of the protein in RPL28 KD sorafenib-resistant cells. **(A)** MHC Class I;**(B)** PD-L1;**(C)** EGFR; **(D)** TRAF6.
