# Correction: RPL28 mediates sorafenib resistance in hepatocellular carcinoma by downregulating CDC6 expression

**DOI:** 10.3389/fonc.2026.1814738

**Published:** 2026-03-04

**Authors:** Yi Shi, Fangfang Chen, Yuanyuan Weng, Hang Zeng, Gang Chen

**Affiliations:** 1 Department of Molecular Pathology, Clinical Oncology School of Fujian Medical University, Fujian Cancer Hospital, Fuzhou, Fujian, China; 2 Department of Pathology, School of Basic Medical Sciences, Fujian Medical University, Fuzhou, Fujian, China

**Keywords:** ribosomal proteins L28 (RPL28), sorafenib resistance, hepatocellular carcinoma, CDC6, transcriptome, proteome

An incorrect **Funding** statement was provided “The author(s) declared that financial support was not received for this work and/or its publication.”. The correct **Funding** statement reads: “This study was supported by Joint Funds for the innovation of science and technology, Fujian province (No. 2021Y9214) and Natural Science Foundation of Fujian province (No. 2023J011259).”

In the abstract, “RPL28 was silenced by siRNA, and effects on cell proliferation, migration, and sorafenib sensitivity were assessed by CCK-8, migration assays, and IC50 determination.” is incorrect. This has been corrected to read: “RPL28 was silenced by siRNA, and effects on cell proliferation, and migration were assessed by CCK-8 and migration assays.”

The original version of this article has been updated.

