# Supplementary figures and images for: RPL28 mediates sorafenib resistance in hepatocellular carcinoma by downregulating CDC6 expression
Source: Front Oncol. 2026 Feb 16;16:1741406. doi: 10.3389/fonc.2026.1741406 (PMC12951193; doi:10.3389/fonc.2026.1741406)

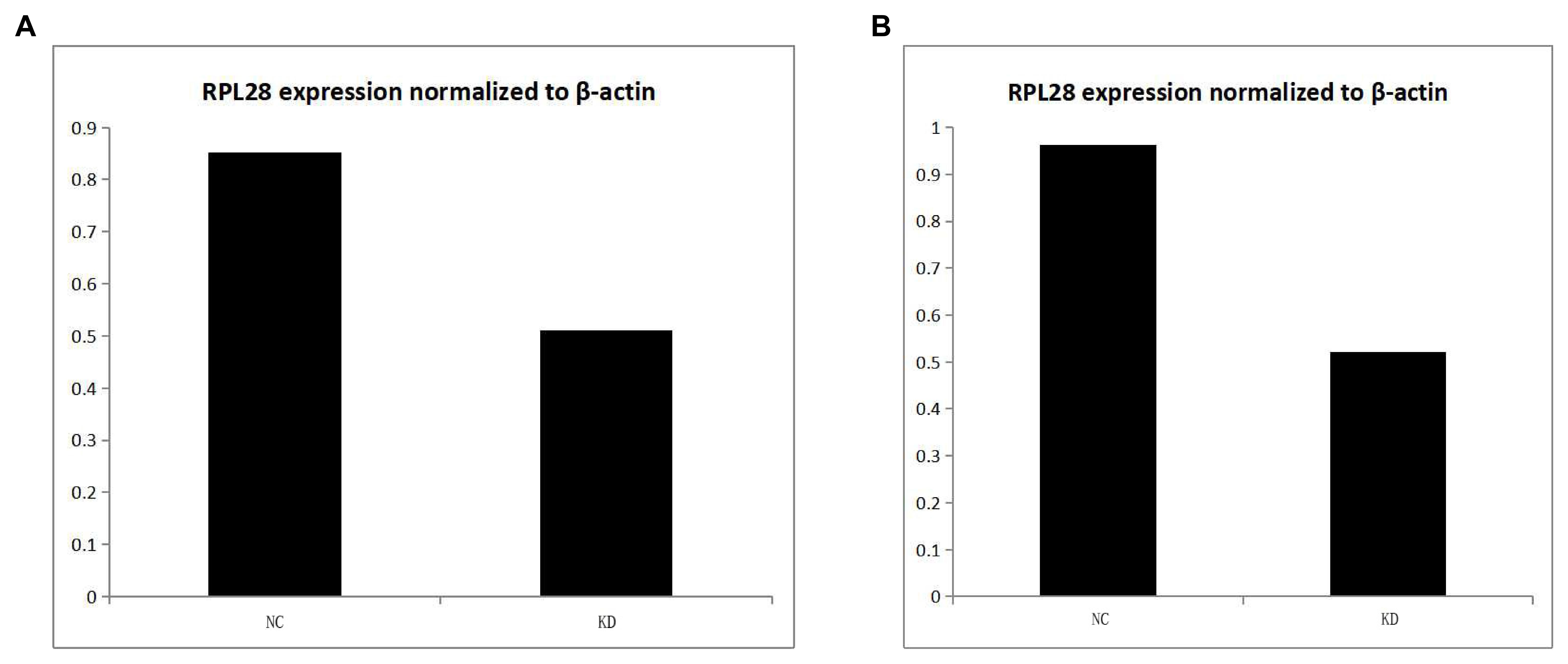

Supplement: Supplementary Figure 1 — The quantification of RPL28 protein. (A) HepG2 KD sorafenib-resistant cells; (B) HCCLM3 KD sorafenib-resistant cells. [file Image1.jpeg]

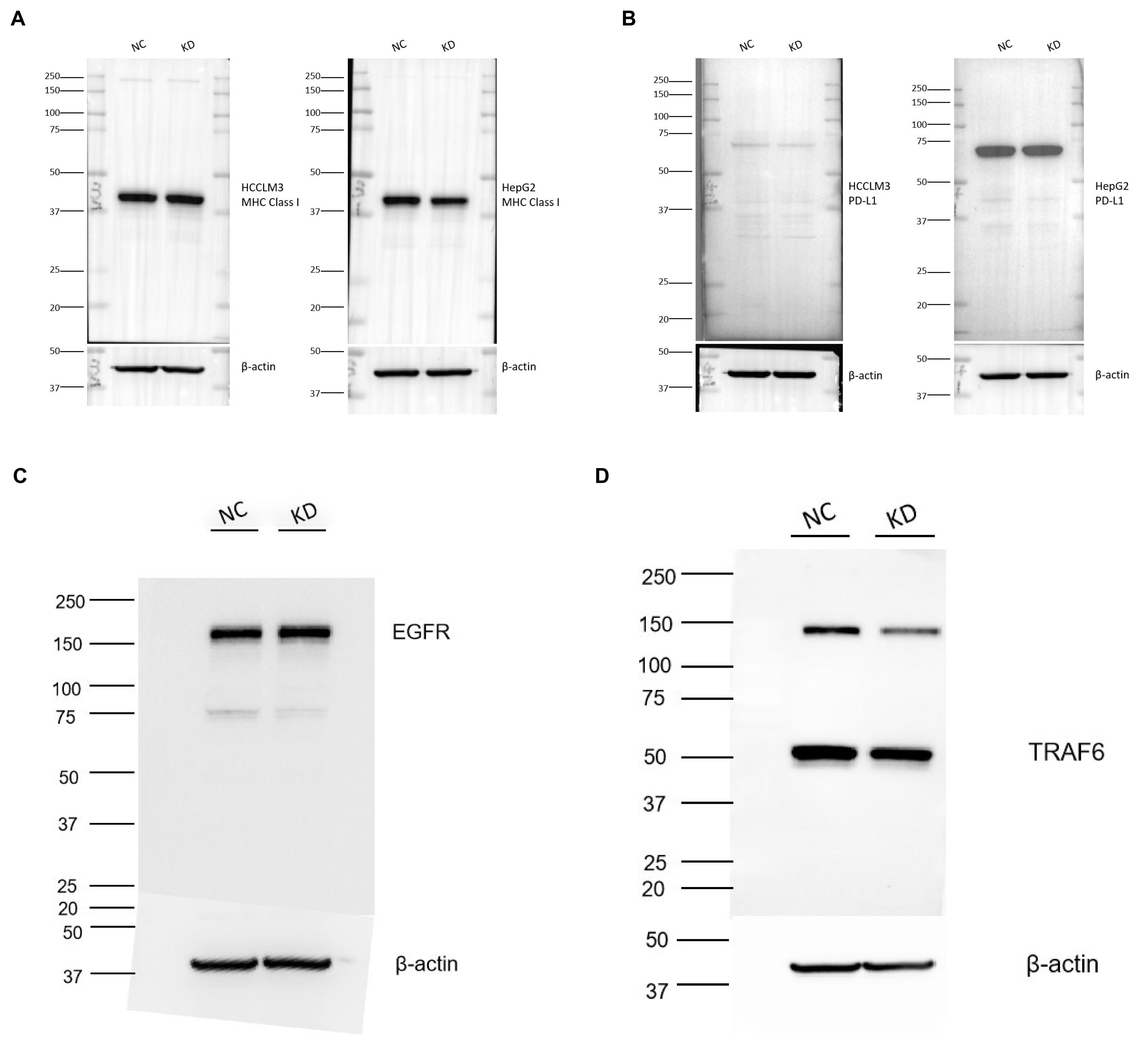

Supplement: Supplementary Figure 2 — Western blot of the protein in RPL28 KD sorafenib-resistant cells. (A) MHC Class I;(B) PD-L1;(C) EGFR; (D) TRAF6. [file Image2.jpeg]
